# Inebilizuma treatment for acute serum AQP4 – IgG neuromyelitis optica: Case report

**DOI:** 10.1097/MD.0000000000045293

**Published:** 2025-10-31

**Authors:** Shou-hui Zhu, Dong-hua Liu, Qian Liu, Qing Chen, Yang Shen, Su-yun Yang, Bing-xin Pan, Fang Yang, Peng-fei Jiang

**Affiliations:** a Ophthalmology Center of Quzhou Hospital of Zhejiang Medical and Health Group, Quzhou, China; b Ophthalmology of Fengdu County People’s Hospital, Fengdu, China; c Nursing of Qujiang Chinese Medicine Hospital, Quzhou, China.

**Keywords:** aquaporin⁃4, Inebilizumab, intravenous methylprednisolone pulse, neuromyelitis optica

## Abstract

**Rationale:**

Neuromyelitis optica spectrum disorder (NMOSD) is a relapsing-remitting, chronic progressive inflammatory demyelinating disease of the central nervous system, predominantly affecting the optic nerves and spinal cord. Currently, there is no effective cure, and the overall prognosis is poor. Notably, the aquaporin-4 immunoglobulin G (AQP4-IgG) seropositive subtype (historically termed neuromyelitis optica) is associated with a significantly worse prognosis.

**Patient concerns:**

Findings included left optic disc edema, prolonged P100 wave latencies and reduced amplitudes on visual evoked potentials (VEP) bilaterally, and concentric constriction of the left visual field. Fundus fluorescein angiography of the left eye revealed hyperfluorescence of the optic nerve. Contrast-enhanced magnetic resonance imaging demonstrated patchy and linear enhancement in the left optic nerve and linear enhancement in the right optic nerve (serum AQP4-IgG positivity confirmed by a tertiary hospital).

**Diagnoses:**

The patient has been diagnosed with AQP4-IgG seropositive NMOSD.

**Interventions:**

The regimen consisted of intravenous methylprednisolone pulse 1000 mg daily for 5 consecutive days, combined with a single intravenous infusion of inebilizumab (brand name: Uplizna; MedImmune Pharma B.V.; specification: 100 mg/vial) at a dose of 300 mg.

**Outcomes:**

At the 1-month follow-up post-treatment, the patient’s left eye visual acuity recovered to 1.0. Fundus examination revealed normal retinal and macular structure bilaterally with resolved optic disc edema. Right eye VEP showed normalized P100 latency and amplitude. Left eye VEP still exhibited prolonged P100 latency and subnormal amplitude. Visual field testing results were normal in both eyes.

**Lessons:**

This case demonstrates that combination therapy with IVMP and inebilizumab during the acute phase significantly promoted visual recovery in this AQP4-IgG seropositive NMOSD patient. This finding enhances our understanding of NMOSD management. The case offers valuable clinical insights for therapeutic strategies in NMOSD. Future studies with larger cohorts are warranted to further evaluate the efficacy of the intravenous methylprednisolone pulse–inebilizumab combination in reducing relapse rates and improving long-term neurological outcomes in NMOSD.

## 1. Introduction

Neuromyelitis optica spectrum disorder (NMOSD) is an inflammatory demyelinating disease of the central nervous system characterized by a relapsing-remitting and chronic progressive course, predominantly affecting the optic nerves and spinal cord. Effective curative treatments are currently lacking, and the prognosis is generally poor. Historically, neuromyelitis optica (NMO) was considered a clinical phenotype of multiple sclerosis (MS). However, the discovery of a specific autoantibody (AQP4-IgG) targeting aquaporin-4 (AQP4) on astrocytic foot processes established NMO as a distinct disease entity separate from MS. In 2015, the International Panel for NMO Diagnosis revised the diagnostic criteria for NMOSD, incorporating classic NMO within the NMOSD spectrum. The criteria further stratified NMOSD into AQP4-IgG positive and negative subtypes, providing a more detailed delineation that significantly expanded the disease spectrum.^[1,2]^

NMO-associated optic neuritis (NMO-ON) and myelitis may occur sequentially or concurrently. In China, NMO-ON represents one of the most prevalent forms of optic neuritis. NMO-ON frequently results in acute and severe visual loss, with the majority of patients experiencing a precipitous decline in visual acuity to counting fingers or even no light perception during the acute phase, portending a poor prognosis. Visual recovery is often incomplete in a subset of patients despite treatment, with blindness rates reported as high as 75%.^[3]^ Herein, we report a case of NMOSD where treatment with high-dose intravenous methylprednisolone pulse (IVMP) therapy combined with Inebilizumab led to significant visual improvement within 1 month, accompanied by normalization of optic nerve findings and visual field testing. The case details are presented below. This study was approved by the Ethics Committee of Quzhou Hospital, Zhejiang Medical and Health Group.

## 2. Case presentation

**Figure 1.**
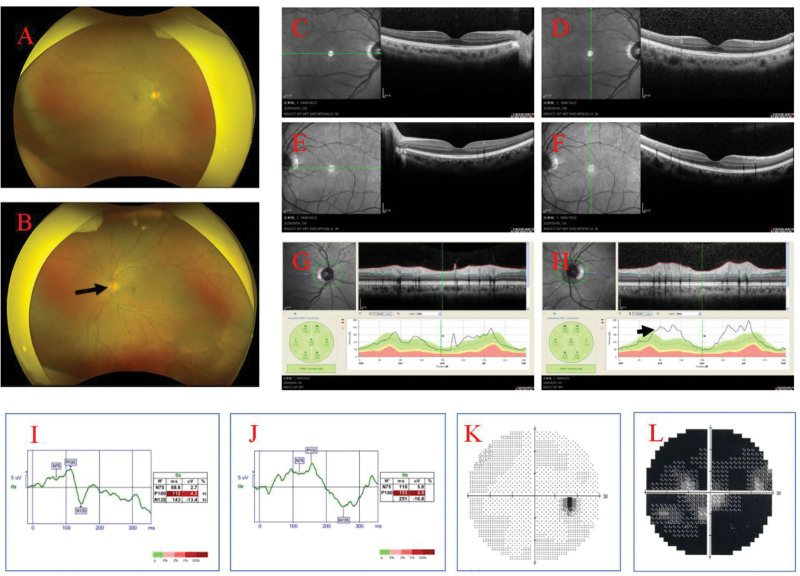
Patient’s initial retinal and visual electrophysiology and visual fields.

**Figure 2.**
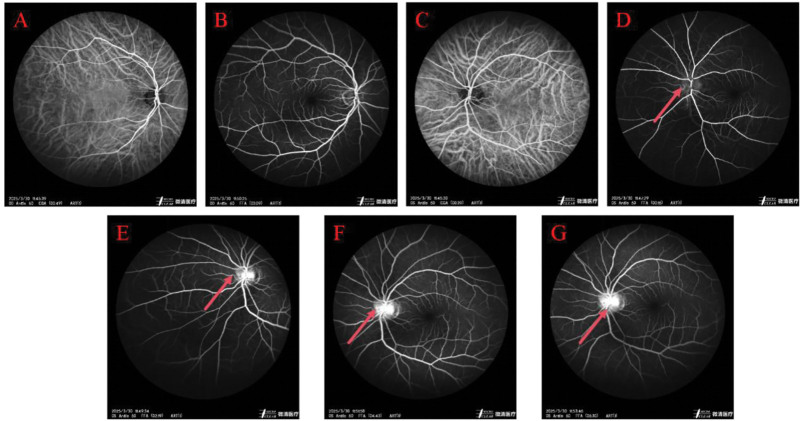
Patient’s initial ICGA and FFA contrast images. FFA = fundus fluorescein angiography, ICGA = indocyanine green angiography.

**Figure 3.**
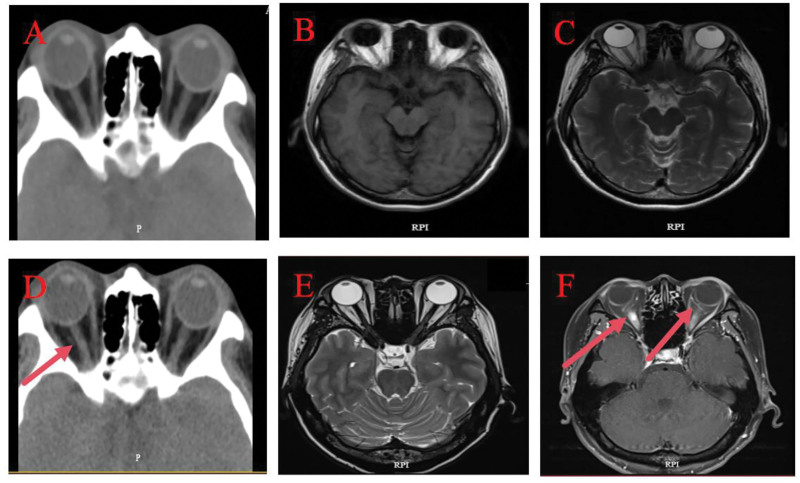
Patient’s initial CT and MRI orbital images. CT = computed tomography, MRI = magnetic resonance imaging.

**Figure 4.**
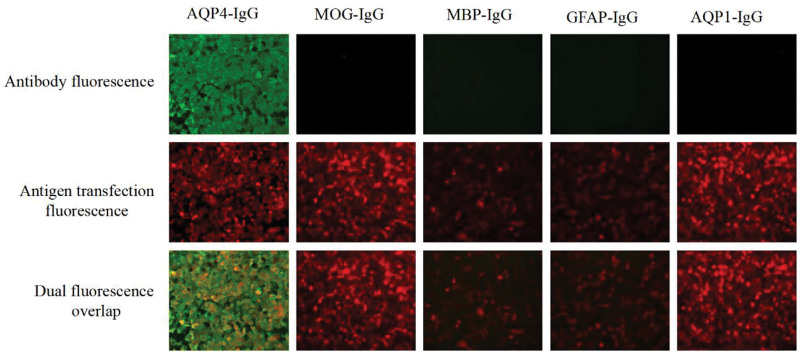
Serum demyelination 5 test in patients.

Patient, female, 36 years. old. presented on March 28, 2025. Complaint: 1 day of vague pain in the left eye. No history of hypertension, diabetes mellitus, no history of surgery, no history of trauma, no history of drug allergy, no history of long-term drug use. Ophthalmological examination: visual acuity of the right eye was 1.2, visual acuity of the left eye was 1.0, intraocular pressure of the right eye was 13 mm Hg, and intraocular pressure of the left eye was 15 mm Hg. Pupils of both eyes had normal light reflex, and refractive interstices were clear. Fundoscopic examination: left eye optic disc boundary is not clear, color is normal, retina is normal, macula is normal. Topical eye drops were used for anti-inflammatory treatment (levofloxacin eye drops.) On March 30, 2025, the patient was seen again. Complaint: left eye pain with vision loss for 2 days. Ophthalmological examination: visual acuity of the left eye was 0.2, visual acuity of the right eye was 1.2. Intraocular pressure of the right eye was 14 mm Hg, and intraocular pressure of the left eye was 12 mm Hg. Fundoscopic examination: the retina of the right eye was clear, with a clear border of the disc and no edema of the disc, while the border of the disc of the left eye was unclear, with an edema of the disc (Fig. 1A and B). optical coherence tomography (OCT) (Spetraslis-X-ray film made by Heidelberg, Germany) was performed on the right eye, and the right eye was examined. Spetraslis-OCT model OCT scanner manufactured by Heidelberg, Germany, with a scanning area of 9.0 mm × 9.0 mm, a scanning mode of Retina 30-degree fundus 9-mm scanning length, a scanning depth of 1.9 mm, an axial resolution of 3.9 μm, and a transverse resolution of 5.9 μm. Examination: the structure of the retina and macula of both eyes was normal, and there was edema of the optic nerve in the left eye (Fig. 1C–H). Visual electrophysiological examination: the P_100_ latency was prolonged and the amplitude decreased in both eyes; the P_100_ latency of the right eye was 112 ms, and the amplitude was 4.3 μV; J: the P_100_ latency of the left eye was 153 ms, and the amplitude was 4.0 μV (Fig. 1I and J). Visual field examination: the visual field of the right eye was normal, and the visual field of the left eye was centripetally narrowed (Fig. 1K and L). indocyanine green angiography (ICGA) and fundus fluorescein angiography examination: fundus fluorescein angiography of the left eye showed strong fluorescence in the optic nerve (Fig. 2). Enhanced magnetic resonance imaging: patchy and striated enhancement was seen in the left optic nerve, and striated enhancement was seen in the right optic nerve (Fig. 3). Diagnosis: NMO. The patient was advised to go to a higher level hospital for AQP4-IgG examination. AQP4-IgG examination: AQP4-IgG antibody was positive, and the rest of the antibodies were negative. Serum AQP4-IgG 1:320 (transfected cell method) (Fig. 4). The diagnosis was acute serum AQP4-IgG NMO.

**Figure 5.**
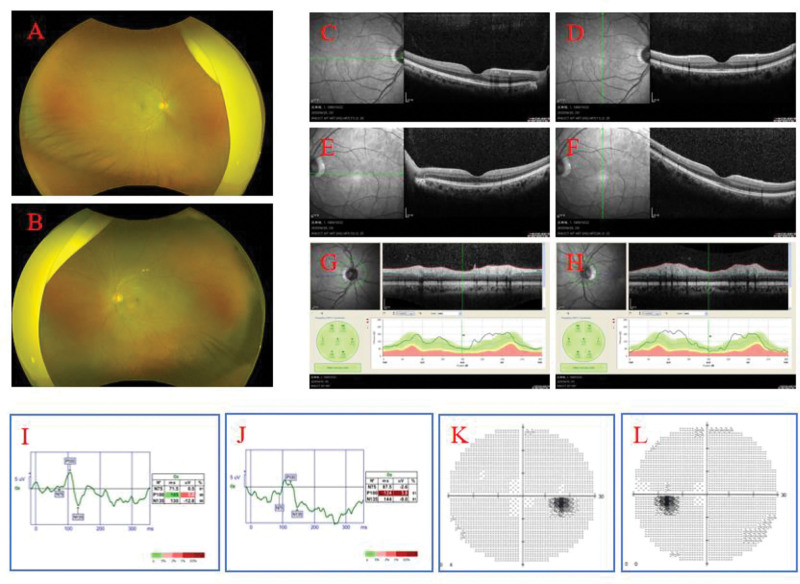
Retina, visual electrophysiology and visual field of patients after treatment.

Patients were treated with IVMP at a dose of 1000 mg for 5 consecutive days. Combined with Inebilizumab (MedImmune Pharma, Netherlands, specification: 100 mg/vial), 300 mg was infused intravenously. The patient was reexamined on April 25, 2025, the visual acuity of the right eye was 1.2, and the visual acuity of the left eye was 1.0. The patient’s visual acuity was stable, and the structure of the fundus retinas and macula was normal in both eyes, and the optic nerve edema had subsided (Fig. 5A–H). Electroretinogram: The P_100_ latency of the right eye was 105 ms, and the amplitude was 5.8 μV, which was normal. In the left eye, the P_100_ latency was still prolonged and the amplitude was still lower than normal, with a P100 latency of 124 ms and an amplitude of 3.8 μV (Fig. 5I and J). Visual field examination: the visual fields of both eyes were normal (Fig. 5K and L).

## 3. Case discussion

Epidemiological studies indicate that NMO-associated optic neuritis (NMO-ON) predominantly affects both eyes, with a higher incidence in middle-aged females (male-to-female ratio ≈ 1:6.1) and an onset age ranging from 16 to 80 years.^[3]^ Standard acute treatments include intravenous methylprednisolone pulse (IVMP) and plasma exchange (PLEX). However, limited plasma supply restricts PLEX availability to tertiary medical centers in China, making IVMP the primary intervention for NMO-ON. IVMP remains the first-line therapy for both acute attacks and recurrent NMO-ON. International guidelines and the Chinese Diagnostic and Therapeutic Guidelines for NMOSD recommend a high-dose corticosteroid regimen: methylprednisolone 1000 mg/day intravenously for 3 to 5 days.^[4]^ Notably, a retrospective analysis demonstrated comparable efficacy between 1000 mg and 500 mg methylprednisolone in acute NMO-ON after adjusting for baseline visual acuity and age.^[3]^ Despite this, the 1000 mg dose remains the clinical standard.

B lymphocytes (BLCs) are pivotal components of adaptive immunity. Differentiating from bone marrow stem cells via CD34 upregulation and B-cell receptor rearrangement, they mature into naïve BLCs, memory BLCs, and antibody-secreting cells (CD38⁺ plasmablasts and CD138⁺ plasma cells) in secondary lymphoid organs. Dysfunctional BLCs disrupt central/peripheral immune tolerance, activating pro-inflammatory subsets and driving pathogenic AQP4-IgG production – a key event in NMOSD pathogenesis. B-cell depleting therapy (BDT) is frequently used in myelin oligodendrocyte glycoprotein antibody-positive (MOG-IgG⁺) NMOSD due to its pathological overlap with MS (e.g., type II demyelination). In contrast, BDT efficacy in AQP4-IgG⁺ NMOSD is less established. While BDT is recommended as first-line relapse prevention,^[5]^ high-level evidence supporting its initial use in acute NMO-ON is lacking.

Inebilizumab is a humanized anti-CD19 monoclonal antibody that depletes CD19-expressing pathogenic B cells – including pro-B cells, mature B cells, memory B cells, and plasmablasts – yielding broad efficacy.^[6]^ In June 2020, it became the first FDA-approved B-cell-targeted therapy for NMOSD.^[7,8]^ Long-term follow-up of 75 AQP4-IgG⁺ patients revealed only 13 relapse events (total 18 attacks) over 4 years of Inebilizumab treatment, with no disability progression and enhanced efficacy by Year 2.^[9]^

Compared with AQP4-IgG⁻ NMO-ON, the AQP4-IgG⁺ subtype causes more severe visual impairment and exhibits poorer treatment response. Studies report ≤ 1-line visual acuity improvement in 50% of IVMP-treated patients,^[3]^ particularly those with baseline vision of counting fingers to 0.3. Our findings indicate that IVMP-BDT combination therapy promotes significant visual recovery and reversal of concentric visual field defects.

While IVMP-Inebilizumab demonstrated short-term benefits in this acute-phase cohort, its long-term impact on relapse reduction remains undetermined due to limited follow-up. Given the cumulative nature of visual damage in NMO-ON (with 80% to 90% experiencing relapsing courses^[3]^), future studies with expanded cohorts and extended follow-up are warranted to evaluate this regimen’s potential in relapse prevention and long-term functional preservation.

## Acknowledgments

The authors sincerely thank the patients for participation in the present study.

## Author contributions

**Data curation:** Qian Liu, Qing Chen, Yang Shen, Su-yun Yang, Fang Yang.

**Funding acquisition:** Peng-fei Jiang.

**Writing – original draft:** Shou-hui Zhu, Dong-hua Liu, Bing-xin Pan.

**Writing – review & editing:** Fang Yang, Peng-fei Jiang.
